# Artefact design and societal worldview

**DOI:** 10.1098/rsta.2024.0092

**Published:** 2024-11-13

**Authors:** Elisabeth Stockinger, Michael Mandlmayr

**Affiliations:** ^1^ Department of Humanities, Social and Political Sciences, ETH Zurich, Zurich 8092, Switzerland; ^2^ Voestalpine Stahl GmbH, Linz 4020, Austria

**Keywords:** computational social science, sociotechnical systems, voting advice application

## Abstract

Technological artefacts are created in accordance with the values and worldviews of their designers. In operation, they act as a medium, facilitating and constraining human interaction with, and perception of, the world. When used on a large scale, they may lastingly affect societal ethos. If institutional structures of domination allocate the resources necessary for artefact design and development to some population groups over others, the direction and extent of such an effect may lead to increased disparity and inequity. While the direct influence of technology on opinion is well-studied, the evaluation of non-epistemic values, assumptions and presuppositions is a hurdle in the way of a deeper understanding of the large-scale effects of asymmetries in worldviews embodied by artefacts. Here, we show that artefacts have a strong potential to bias societal worldviews when they are distributed unevenly across the value spectrum. They can affect the clustering behaviour of agents with regard to worldview, both aiding and hindering intra- and inter-cluster diversity, depending on their distribution and frequency. Our findings underline the distributional sensitivity of worldview dynamics to institutional structures of domination. We highlight the importance of procedural interventions such as participatory design, which explicitly acknowledges existing asymmetries and redistributes power accordingly.

This article is part of the theme issue ‘Co-creating the future: participatory cities and digital governance’.

## 1. Introduction

Artefacts of human design—be they products of engineering, art, or thought—modulate our understanding of the world, our decision-making processes, and the way we organize as a society.

In particular, digital technology shapes and reshapes modern social and societal structures [1], affecting and changing the social contexts into which it is introduced [2,3]. Technological artefacts must, therefore, be held to high normative standards to prevent a harmful or adverse contribution to societal development.

Juridical efforts of asserting such bounds include fairness and ethics legislation such as the AI Act [4] or the GDPR [5]. In particular, much attention is given to human oversight, algorithmic transparency and explainability. However, most artefacts are contained within a greater context including use case and human interaction, environmental factors and feedback loops. Social concepts such as fairness or equity are procedural, contextual and contestable, and cannot fully be accounted for in mathematical formalism [2].

Ehsan *et al*. [6] call for a higher valuation of social, organizational and cultural factors in transparency research and evaluation. Wagner [7] criticizes the common set-up of human-in-the-loop systems as a way of rubber-stamping an otherwise completely automated decision-making system, rather than asserting meaningful human agency. Similarly, Rudin [8] promotes a shift towards inherently interpretable models for high-stake decisions to enable meaningful human oversight.

Beyond these challenges to the formal evaluability of non-epistemic requirements and to regulatory efforts, Mohamed *et al*. [1] argue that technological and, in particular, AI systems must be understood in their historic context of coloniality and power imbalance.

Explicitly or implicitly, artefacts are shaped according to the values and perspectives of those involved in the design process [9–11]. Through repeated interaction, they may in turn propagate their constituent worldview to their users [12]. A worldview, here, is a person’s explanatory mental model of the world, including values, opinions, attitudes and expectations, and directly informs his or her actions [13]. Some proponents of critical theory consider top-down value definitions as are common in regulation to be of colonial character, disregarding and invading minority or local value contexts [1]. Imposing normative values through principalist ethics frameworks has been considered an imposition by the powerful, bearing the risk of precluding alternative visions [14].

In this article, we investigate the effect of representativity of population worldviews by large-scale technological artefacts on societal value distributions. In particular, we study the clustering behaviour according to opinions and values, the biasing of the societal worldview distribution in favour of subgroups, and the openness towards viewpoints different from one’s own.

## 2. Structurational model of technology

**Figure 1.**
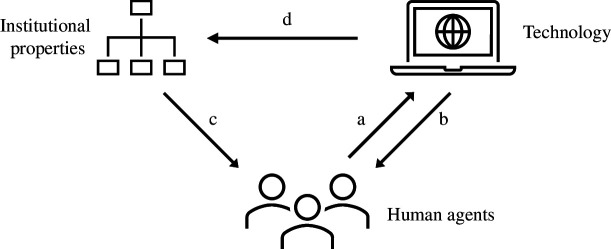
Structurational model of technology as presented by Orlikowski [15]. Each arrow indicates directional influence during design and development or during utilization of technology.

We draw on the structurational model of technology by Orlikowski [15] based on Giddens theory of structuration [16] to inform our conception and model of the effect of artefacts across a society. This model formalizes the interaction and influence between (i) human agents such as technology designers, users and decision-makers, (ii) technological artefacts mediating human action and (iii) institutional properties, including organizational dimensions such as ideology, culture and control mechanisms (figure 1).

Fundamentally, technology is the product of human action (arrow a): it is constructed physically by actors working in a given social context, and socially through the meaning that actors attach to it and by the features that are emphasized and used. Once deployed, technology facilitates and constrains human action either directly through the use cases and solutions it supports, or indirectly through the mental models or norms it embodies (arrow b). Institutional properties enact influence on designers and developers of technology, for example, by defining goals and interfaces, and on users in their interaction with technology, for example, by establishing standards, providing resources or guiding intentions (arrow c). Finally, technology assumes structural properties: once developed and deployed, technology may reinforce or transform institutional structures of signification (representing the organizational rules informing and defining interactions and extending to shared knowledge), of domination (reflecting asymmetries in the potential of agents to enact change) and of legitimation (articulating and sustaining moral order through tradition of socialization practices, arrow d) [15].

The structurational model [15] limits its scope of technology to material artefacts, defined as various configurations of hardware and software. As our article focuses on the societal impact of artefacts, we further restrict our scope towards artefacts that are available to most stakeholders within a society, and that are used for the formation and exchange of opinions. We therefore include, for example, social-media networks, newspaper websites and aggregators and specific-purpose applications such as voting advice applications (VAAs). We exclude, on the other hand, business-internal websites targeting or accessible to only a small subset of a population, or applications not involved in opinion formation such as games or office tools.

The use and development of artefacts typically involve different sets of human agents at different points in time. Designers and developers, influenced by the institutional properties present at the time, fashion technology to meet managerial or personal goals (arrows c and a). Only at a later point does the artefact enact influence on its users and on institutional properties (arrows b and d). Artefacts remain modifiable throughout their life cycle, with both users and designers able to potentially change the artefact subsequently. This human action will then be influenced by the potentially changed institutional properties.

We here illustrate the application of the structurational model on the example of VAAs [17], interactive tools intended to aid the user in choosing a party or candidate to vote for in an upcoming election.^1^ These applications map the political space pre-election with a questionnaire. The distance between users’ and candidates’ answers to the questionnaire is compared and sorted, and candidates are recommended accordingly. VAAs are technological artefacts created by technology designers under the influence of the institutional properties of domination, signification and legitimation. Structures of domination allocate the power and resources to shape the VAA to certain population groups over others. VAAs are often subsidized publicly, giving current governing actors a hand in VAA design directly by including public officials in development, or indirectly in the funding of actors involved therein. Institutional structures of signification include the rules informing and defining the users’ interpretation of the relevancy of factors and specific issues in making an electoral choice. VAAs necessarily emphasize certain issues and trade-offs over others and by design single out political alignment as the sole factor in their recommendation, thwarting possible considerations such as perceived qualities of leadership or honesty. Structures of legitimation include those practices sustaining moral order within a society. VAAs embody an ideological and normative perspective on democracy and on the forms of competence required for good citizenship [18,19]. In particular, these presuppositions are associated with the social choice conception of democracy [18], where user preferences are considered static and the core mission is preference aggregation. These are contestable and contingent commitments framing the design of the VAA.

Once deployed, VAAs interact with human agents through utilization. They constrain the users’ search for an electoral recommendation through the restriction to political alignment as the factor of relevance and through the selection of political issues included in the application. While users remain free and are often encouraged to seek for alternative sources of information, the clear framework of coming towards an electoral choice may further institutionalize the structural legitimization of social choice model of democracy as well as the norms and values prioritized within a society.

Social-media services such as X or Facebook hold similar normative characters, shaped by the institutional properties of the companies developing them, emphasizing the importance of engagement and popularity over consensus and deliberation.

## 3. Bounded confidence model in the presence of artefacts

The structurational model illustrates the potential of technological artefacts to have a lasting effect on users in their interpretation and conception of the world, and, in the longer term, on societal norms and structures. We focus on the effect of asymmetric structures of domination granting certain groups greater resources and power over others at the design stage (figure 1, arrow a) and the subsequent effect on user worldviews at the utilization stage (arrow b). This allows us to treat artefacts as static and shifts the focus to the effect of technology on human agency.

Given a society in the continuous process of political orientation and formation of governance, we consider the following non-epistemic concepts involved in human agency:

α: normative democratic values, and
β: opinions within political issues such as social welfare, migration policy, etc.

Following critical theory and value-based design, artefacts inherently contain the worldview of their designers [9,10,12,20]. Based thereon, we assume that regulation may avoid bias on the level of opinion but cannot oversee the values an artefact propagates. Normative democratic values will affect concrete opinions towards political issues. For example, a citizen valuing deliberation, open-mindedness and the public sphere may support public funding of independent journalism to a greater extent than another citizen who puts greater emphasis on autonomy and self-development. We assume a one-directional influence of normative values on to political opinions. This is a simplification for the sake of interpretability. There may exist a two-directional coupling whereby political decisions affect the real world and, in turn, the worldview of the citizen. We now look into the possible effect of the external forcing of values (inspired by Brooks and Porter [21]) through artefacts on societal value distributions.

We use a bounded confidence model inline with the work of Hegselmann and Krause [22–24], where society is represented as a social network graph $G(\nu_{N},e)$. Each node (or agent) in the set $\nu_{N}$ represents a citizen. Each agent has a certain worldview, here simplified to a position along two axes (normative democratic values α and concrete political opinions β). The agents may change their positions along these axes over time. At the discrete time step t, an agent i’s worldview is given as ${𝐱}_{i}^{t}=(\alpha ,\beta )\in [0,1{]}^{2}$. Agents exchange their values and opinions with one another. Each edge in the set e represents an observation of one agent’s values and opinions by another. Specifically, agent i observes agent j’s behaviour if there is a directed edge from j to i.

We now introduce M artefacts within this network. We assume that artefacts are observable by all agents and that their positions on the value space are immutable. Therefore, the in-degree of a technological artefact is always 0 while the out-degree is N. We assume the idealized case where regulation completely removes influence on concrete opinion such that artefacts have no β value.

**Locality**. We can represent this social network in a constant adjacency matrix A representing process locality, where $A_{ij}=1$ if agent i may observe an action by another agent j at a given time step. If $A_{ij}=0$, agent i will never observe an action by agent j. We therefore define the adjacency matrix $A\in \{0,1{\}}^{\left(N+M)\times (N+M\right)}$ as


(3.1) $$\begin{array}{r} A=\left[\begin{array}{cc} A_{ag,ag} & A_{ag,ar}\\ A_{ar,ag} & A_{ar,ar} \end{array}\right]=\left[\begin{array}{cc} A_{ag,ag} & 1\\ 0 & 0 \end{array}\right], \end{array}$$

where $A_{ag,ag}\in \{0,1{\}}^{N\times N}$ are the connections between the agents, $A_{ag,ar}\in \{0,1{\}}^{N\times M}=𝟙$ are the edges from the agents to the artefacts, $A_{ar,ag}\in \{0,1{\}}^{M\times N}=𝟘$ are the edges from the artefacts to the agents, $A_{ar,ar}\in \{0,1{\}}^{M\times M}=𝟘$ are the edges between the artefacts, and $A_{ii}=1$. As artefacts cannot communicate with each other or observe agents, the last M rows are discarded.

**Receptivity**. Agents are influenced by other nodes in their surroundings and on each time step t synchronously adjust their worldview to reflect this ($\alpha^{t+1}=f(\alpha^{t})$ and $\beta^{t+1}=g(\alpha^{t},\beta^{t})$). Agents will only be receptive to nodes with worldviews sufficiently close to their own. This notion is defined by a confidence interval of size ϵ. We define receptivity by the variable matrix R(𝐱) of the same dimension as A:


(3.2) $$R_{ij}(x^{t})=I(dist(x_{i}^{t},x_{j}^{t})<\epsilon ),$$

where I is the indicator function and returns 1 if the operand is true and 0 otherwise.

**Update rule**. Agents update their normative values α by averaging the values of their neighbours to which they are receptive. Opinion updates β consider both opinions and values.

As we assume the idealized case where artefacts have no effect on user opinions, they play no role in the β update. They do, however, contribute to the agent’s α updates. Therefore agent i has two different sets of neighbours N where $N^{\beta }\subset N^{\alpha }$:


(3.3) $$\begin{array}{rl} N_{i}^{\alpha }(x^{t}) & =\{j\in 1,...,N+M|\;A_{ij}R_{ij}(x^{t})=1\}, \end{array}$$

(3.4) $$\begin{array}{rl} N_{i}^{\beta }(x^{t}) & =N_{i}^{\alpha }(x^{t})\cap \{1,\ldots ,N\}, \end{array}$$

At time step t, agent i updates its 𝐱 values as follows:


(3.5) $$\begin{array}{rl} \alpha_{i}^{t+1} & =\frac{1}{|N_{i}^{\alpha }(x^{t})|}\sum_{j\in N_{i}^{\alpha }(x^{t})}\alpha_{j}^{t}\\ & =AVG\;\left(\left\{\alpha_{j}^{t}|\;j\in N_{i}^{\alpha }(x^{t})\right\}\right),\\ \beta_{i}^{t+1} & =\frac{1}{|N_{i}^{\beta }(x^{t})|}\sum_{j\in N_{i}^{\beta }(x^{1})}\left(\lambda \beta_{j}^{t}+(1-\lambda )\alpha_{j}^{t}\right)\\ & =\lambda AVG\left(\left\{\beta_{j}^{t}|\;j\in N_{i}^{\beta }(x^{t})\right\}\right)+(1-\lambda )AVG\;\left(\left\{\alpha_{j}^{t}|\;j\in N_{i}^{\beta }(x^{t})\right\}\right), \end{array}$$

where λ∈[0,1] is a tradeoff term defining the extent of ideological influence on concrete political opinions.

For a fixed $N^{\alpha }$ and $N^{\beta }$, this averaging operation is linear and can be described by matrix A. Hence, for every 𝐱 there exist $A^{\alpha }(x)$ and $A^{\beta }(x)$ such that


(3.6) $$\begin{array}{rl} F(x^{t}) & :=\left\{ \begin{array}{ll} \alpha_{ag}^{t+1} & =A^{\alpha }(x^{t})\alpha^{t}\\ \beta_{ag}^{t+1} & =\lambda A^{\beta }(x^{t})\beta^{t}+(1-\lambda )A^{\beta }(x^{t})\alpha^{t}\\ & =A^{\beta }(x^{t})(\lambda \beta^{t}+(1-\lambda )\alpha^{t}). \end{array}\right. \end{array}$$

**Social network structure**. Influence and information propagation within a social network depend strongly on its structure. We use a directed social network graph with reciprocal and directed edges based on $\chi^{2}$-distributed degree correlation following the graph generation method described by [25]. This includes a high clustering coefficient with small average distances between random node pairs and is topologically and algorithmically similar to real-world graphs.

Within our simulations, we use a network of 10 000 nodes, a Spearman’s rank correlation of 0.4 between reciprocal and in-degrees, of 0.5 for reciprocal and out-degrees, and of 0.3 for in- and out-degrees. The degrees are sampled from a $\chi^{2}$ distribution with a shape parameter of 0.5, location 0.49 and scale 20. For details on implementation and hyperparameter choices, we refer to Schweimer *et al.* [25].

**Simulation configuration**. We use the aforedescribed model to investigate the convergence of opinion and value distributions within a society into equilibria states over time under different initial artefact distributions. Specifically, we investigate the following conditions:

- *not influenced* by artefacts,
- containing *only three* artefacts evenly distributed over the value space,
- containing ten artefacts *evenly distributed* over the value space,
- containing artefacts over the whole value space, but *biased* in frequency towards one pole, and
- containing artefacts *shifted* towards one pole of the value space.

The number and distribution of artefacts must not be considered in isolation. Their effect depends highly on the value of ϵ. A higher openness in a population to consider diverging opinions (represented by a larger ϵ) sensitizes the model to the introduction of artefacts owing to their increased reach. Therefore, we run each condition for ϵ∈[0.05,0.06,…,0.3].

For every ϵ, 𝐱 is sampled uniformly from [0,1) and used to initialize each condition. Each simulation is run on the same static social network A until convergence with a tolerance of $10^{-6}$. All simulations converged after at most 5800 steps. We refer to worldview at the last time step of a simulation as ${𝐱}^{n}\to \bar{𝐱}$.

## 4. Dynamics

We identify the following major areas of impact of artefacts:

— a bias in agent worldview,
— a behavioural change to worldview clustering on a societal level, and
— a qualitative change to the emerging clusters.

**Intended bias in agent worldview under conditions of monopoly**. Within the bounded confidence model, artefacts can be abused to purposefully manipulate societal worldview. This external forcing is trivial under conditions of monopoly. Consider a function describing the value of artefact i at time step t intending to manipulate the societal value distribution towards $\alpha^{*}$:


(4.1) $$f_{i}^{\alpha^{*}}(t)=(1-\Lambda (t))\left(\frac{2i-M}{M}\right)+\Lambda (t)\alpha^{*},$$

where $\Lambda :{\mathbb{R}}^{+}\mapsto \left[0,1\right]$ with Λ(0)=0 and $\lim_{x\to \infty }\;\Lambda (x)=1$, monotonically increasing, describes the speed to change artefact values. The derivative of Λ must not be too large, as agents need time to converge towards the artefact-biased values. Overly fast change may remove artefacts from the agents’ ϵ range. The maximum speed depends mainly on the number of artefacts M and the communication threshold ϵ.

**Figure 2.**
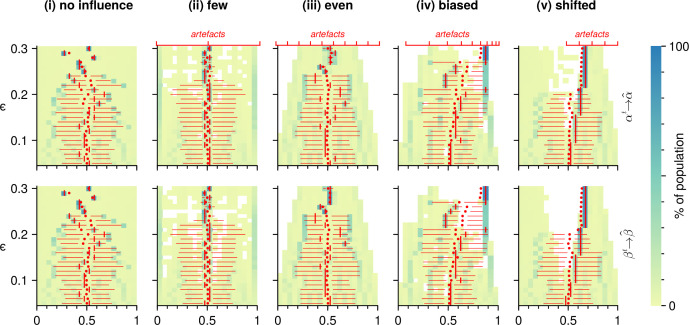
The distribution of α (top) and β values (bottom) upon completion of simulations with different artefact distributions (columns). Each row within a panel represents the result after one simulation run with the ϵ-value specified on the *y*-axis. The colour encodes the ratio of the population value within α or β intervals of width 0.05. White areas are not populated by any agents. Artefact value positions for the given condition are indicated at the top axis and are only defined for α. The horizontal bars within each panel represent the interquartile range, the vertical dashes the median and the small dots the mean α and β values.

**Bias under conditions of competition**. The potential of artefacts to create bias also holds in conditions of competing but imbalanced worldviews when the relationship of ϵ to artefact distribution is sufficient. Figure 2 shows the different resulting value distributions after simulation runs under the five conditions over a range of ϵ values. At lower ϵ values, the differences are small: there are agents populating the entire breadth of the worldview space, and the mean α and β values are approximately centred. However, as ϵ increases, the distributions of 𝐱-values are significantly affected by artefacts (table 1). In particular when artefacts are biased or shifted towards a given direction, the resulting distribution shift is notable already in simulations with low ϵ-values (table 1, columns <biased and <shifted).

**Table 1.** Kruskal–Wallis H test results for the alternative hypothesis that β samples for the different conditions do not originate from the same distribution, as well as Mann–Whitney U test results for the alternative hypothesis that β-distributions completion of simulations affected by artefacts are not equal to simulations not including artefacts. For biased and shifted conditions, the p-value for a one-tailed test with the alternative hypothesis that the distributions underlying 𝐱 are greater than they are in conditions without artefact influence is given in addition.

ϵ	Kruskal–Wallis		no influence & few		no influence & even		no influence & biased			no influence & shifted		
	H	p-value	U	p-value	U	p-value	U	p-value	<biased	U	p-value	<shifted
0.05	4	0.396	4 98 88 701	0.785	5 04 40 487	0.281	4 93 20 046	0.096	**0.048**	5 02 83 660	0.487	0.756
0.06	8	0.089	5 05 39 510	0.186	4 98 25 301	0.669	4 98 76 228	0.762	0.381	4 98 27 652	0.673	0.336
0.07	12	**0.021**	4 87 44 256	**0.002**	4 96 26 437	0.360	4 93 09 938	0.091	**0.045**	4 82 44 410	**<0.001**	**<0.001**
0.08	38	**<0.001**	5 08 75 434	**0.032**	4 93 34 524	0.103	4 89 68 129	**0.011**	**0.006**	4 85 44 194	**<0.001**	**<0.001**
0.09	60	**<0.001**	4 85 99 674	**<0.001**	4 98 09 171	0.640	4 81 15 007	**<0.001**	**<0.001**	4 76 87 082	**<0.001**	**<0.001**
0.10	192	**<0.001**	5 04 59 354	0.260	5 22 75 456	**<0.001**	4 66 58 641	**<0.001**	**<0.001**	5 01 91 434	0.639	0.680
0.11	81	**<0.001**	4 90 06 449	**0.015**	4 95 51 591	0.272	4 71 31 939	**<0.001**	**<0.001**	4 82 59 576	**<0.001**	**<.001**
0.12	57	**<0.001**	5 06 70 050	0.101	4 78 55 010	**<0.001**	4 83 89 641	**<0.001**	**<0.001**	4 67 27 978	**<0.001**	**<0.001**
0.13	129	**<0.001**	4 78 32 743	**<0.001**	5 43 77 838	**<0.001**	4 71 87 933	**<0.001**	**<0.001**	4 93 70 798	0.123	0.062
0.14	69	**<0.001**	4 74 33 273	**<0.001**	5 06 15 222	0.132	4 90 68 428	**0.023**	**0.011**	4 83 60 872	**<0.001**	**<0.001**
0.15	631	**<0.001**	5 07 96 499	0.051	5 27 94 585	**<0.001**	4 07 18 236	**<0.001**	**<0.001**	4 95 32 495	0.252	0.126
0.16	740	**<0.001**	5 32 34 193	**<0.001**	5 37 52 580	<**0.001**	4 02 70 413	**<0.001**	**<0.001**	4 47 11 492	**<0.001**	**<0.001**
0.17	435	**<0.001**	5 15 66 596	**<0.001**	5 98 31 043	**<0.001**	4 71 16 795	**<0.001**	**<0.001**	5 80 20 705	**<0.001**	1.000
0.18	949	**<0.001**	5 17 71 357	**<0.001**	3 66 00 687	**<0.001**	4 01 91 866	**<0.001**	**<0.001**	5 37 78 362	**<0.001**	1.000
0.19	993	**<0.001**	4 99 84 903	0.971	4 67 58 457	**<0.001**	3 79 12 973	**<0.001**	**<0.001**	5 04 73 244	0.246	0.877
0.20	496	**<0.001**	5 18 78 421	**<0.001**	5 38 35 795	**<0.001**	4 55 52 151	**<0.001**	**<0.001**	5 81 66 415	**<0.001**	1.000
0.21	3818	**<0.001**	5 22 66 093	**<0.001**	3 53 23 000	**<0.001**	3 26 59 381	**<0.001**	**<0.001**	1 76 70 085	**<0.001**	**<0.001**
0.22	1727	**<0.001**	5 00 61 106	0.881	6 48 18 180	**<0.001**	3 34 74 881	**<0.001**	**<0.001**	4 81 30 254	**<0.001**	**<0.001**
0.23	1889	**<0.001**	4 57 10 936	**<0.001**	6 41 64 720	**<0.001**	3 06 23 652	**<.001**	**<0.001**	4 60 20 750	**<.001**	**<0.001**
0.24	3927	**<0.001**	4 40 70 052	**<0.001**	5 21 03 307	**<0.001**	3 08 86 528	**<0.001**	**<0.001**	1 95 77 894	**<0.001**	**<0.001**
0.25	16 383	**<0.001**	2 44 26 187	**<0.001**	6 14 67 259	**<0.001**	1 72 64 450	**<0.001**	**<0.001**	1 80 62 346	**<0.001**	**<0.001**
0.26	18 001	**<0.001**	2 22 31 596	**<0.001**	5 49 61 195	**<0.001**	1 53 24 336	**<0.001**	**<0.001**	1 71 80 593	**<0.001**	**<0.001**
0.27	10 290	**<0.001**	2 19 41 050	**<0.001**	1 75 13 244	**<0.001**	4 40 46 693	**<0.001**	**<0.001**	1 57 42 412	**<0.001**	**<0.001**
0.28	22 698	**<0.001**	7 85 49 002	**<0.001**	8 11 44 948	**<0.001**	1 29 93 313	**<0.001**	**<0.001**	1 56 95 436	**<0.001**	**<0.001**
0.29	27 014	**<0.001**	2 07 05 014	**<0.001**	1 56 96 530	**<0.001**	53 63 601	**<0.001**	**<0.001**	1 46 42 767	**<0.001**	**<0.001**
0.30	23 838	**<0.001**	7 99 44 773	**<0.001**	8 02 52 969	**<0.001**	1 25 66 820	**<0.001**	**<0.001**	1 44 85 782	**<0.001**	**<0.001**

**Spatial clustering**. For all simulations including artefacts, the coverage of the value space by agents decreases with higher ϵ values (figure 2; table 2).

**Table 2.** After simulation runs for a given condition and ϵ-value: mean and standard deviation of 𝐱, as well as ratio of occupied space when binning into a grid of 100 × 100 bins.

ϵ	no influence			few			even			biased			shifted		
	occupied	μ	σ	occupied	μ	σ	occupied	μ	σ	occupied	μ	σ	occupied	μ	σ
0.05	1.00	0.500	0.291	0.86	0.505	0.298	0.53	0.503	0.294	0.71	0.507	0.294	0.76	0.503	0.294
0.06	1.00	0.506	0.283	0.89	0.503	0.293	0.75	0.502	0.291	0.74	0.505	0.292	0.81	0.506	0.287
0.07	1.00	0.499	0.279	0.85	0.505	0.298	0.99	0.497	0.288	0.71	0.505	0.290	0.83	0.505	0.283
0.08	1.00	0.505	0.281	0.80	0.504	0.302	1.00	0.504	0.287	0.63	0.513	0.293	0.93	0.514	0.274
0.09	1.00	0.493	0.280	0.80	0.499	0.309	0.95	0.498	0.286	0.73	0.512	0.298	0.97	0.504	0.287
0.10	1.00	0.500	0.283	0.82	0.499	0.304	0.93	0.489	0.281	0.60	0.532	0.301	0.91	0.507	0.276
0.11	1.00	0.490	0.275	0.73	0.487	0.314	0.93	0.496	0.277	0.70	0.515	0.303	0.93	0.507	0.276
0.12	1.00	0.492	0.274	0.71	0.501	0.318	0.88	0.496	0.279	0.75	0.519	0.300	0.87	0.503	0.277
0.13	1.00	0.513	0.296	0.72	0.512	0.340	0.90	0.494	0.276	0.58	0.542	0.305	0.79	0.511	0.270
0.14	1.00	0.514	0.287	0.75	0.501	0.337	0.88	0.502	0.269	0.63	0.544	0.307	0.77	0.510	0.275
0.15	1.00	0.512	0.262	0.65	0.507	0.354	0.89	0.496	0.267	0.66	0.588	0.257	0.79	0.535	0.260
0.16	1.00	0.505	0.280	0.77	0.498	0.343	0.85	0.496	0.259	0.80	0.555	0.283	0.75	0.535	0.278
0.17	1.00	0.519	0.269	0.72	0.496	0.348	0.78	0.499	0.251	0.64	0.566	0.298	0.72	0.513	0.231
0.18	1.00	0.472	0.254	0.49	0.494	0.357	0.78	0.493	0.241	0.70	0.564	0.298	0.76	0.508	0.243
0.19	1.00	0.483	0.283	0.45	0.499	0.349	0.77	0.501	0.239	0.62	0.599	0.252	0.61	0.522	0.233
0.20	1.00	0.513	0.264	0.51	0.496	0.358	0.76	0.502	0.234	0.64	0.581	0.317	0.68	0.522	0.220
0.21	1.00	0.435	0.232	0.44	0.489	0.353	0.74	0.508	0.240	0.59	0.584	0.322	0.53	0.604	0.161
0.22	1.00	0.553	0.297	0.56	0.499	0.354	0.71	0.501	0.243	0.44	0.648	0.243	0.55	0.641	0.172
0.23	1.00	0.542	0.240	0.45	0.526	0.334	0.65	0.495	0.231	0.46	0.641	0.217	0.53	0.636	0.181
0.24	1.00	0.480	0.221	0.33	0.537	0.310	0.64	0.488	0.229	0.35	0.641	0.211	0.48	0.639	0.178
0.25	1.00	0.498	0.170	0.28	0.518	0.291	0.65	0.496	0.129	0.33	0.672	0.195	0.46	0.643	0.176
0.26	1.00	0.461	0.168	0.57	0.516	0.277	0.66	0.463	0.131	0.36	0.671	0.197	0.43	0.643	0.174
0.27	1.00	0.453	0.157	0.36	0.492	0.252	0.61	0.531	0.129	0.39	0.603	0.264	0.41	0.645	0.168
0.28	1.00	0.571	0.168	0.35	0.496	0.244	0.52	0.533	0.115	0.27	0.800	0.175	0.51	0.646	0.169
0.29	1.00	0.336	0.174	0.50	0.489	0.229	0.53	0.534	0.111	0.17	0.809	0.152	0.39	0.651	0.163
0.30	1.00	0.511	0.157	0.54	0.502	0.216	0.54	0.500	0.103	0.12	0.808	0.151	0.36	0.654	0.158

With sufficiently many evenly distributed artefacts, the decrease is symmetric along the *x*-axes with no agents holding extreme opinions or values in either direction. In the case of few artefacts located at the extreme values poles and centrally, there is a loss of coverage of the moderate left and right. However, the resulting distribution is spread more widely with large clusters at the extreme poles where the artefacts are located. When artefacts are biased or shifted, there is a loss of coverage in the centre of the worldview space. In biased conditions, higher ϵ values lead to a large cluster of agents holding a worldview near the artefact average. Similarly, on the poles with fewer artefacts, agents spread over a space relatively central from the convergence points. When artefacts are shifted, there is a clear tendency towards polarization, with a minority of agents congregating around extreme values.

We study this behaviour using Ripley’s K function [26], which describes the number of points within a given radius of another point. For the worldview vector 𝐱, the sample-based estimate of the K-function for a radius r and its variance normalized version (referred to as H-function [27,28]) are defined as


(4.2) $$\begin{array}{rl} \hat{K}(r) & =\sum_{i\neq j}\frac{I(dist(x_{i},x_{j})<r)}{N}, \end{array}$$

(4.3) $$\begin{array}{rl} \hat{H}(r) & =\sqrt{\frac{\hat{K}(r)}{\pi }}-r. \end{array}$$

**Figure 3.**
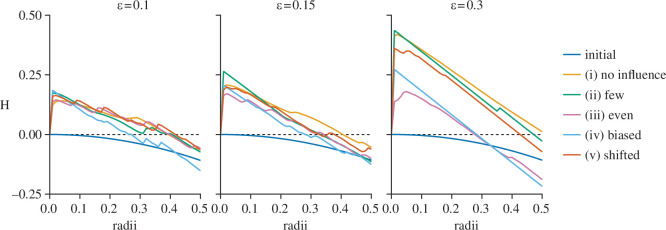
Sample-based estimates of H-values [27] over the simulations of the five different conditions as well as the initial *x*-values with three selected ϵ-values.

Figure 3 shows the H-values over selected simulation runs and search radii as well as for the initial pre-simulation *x*-values. Data following a homogeneous Poisson process would have an H-value of approximately zero, indicated by the dashed black line. All simulations show a high slope of H on short distances across all studied configurations of artefact distributions and ϵ-values, reflecting a high tendency to create clusters of closely spaced values. Similarly, their H-values drop to below zero at higher radii, indicating cluster separation at higher distances. Both the early slope and subsequent drop are more pronounced for larger values of ϵ.

The formation of clusters holding nearly identical values is most pronounced for simulation runs affected by no or by few artefacts. H-values for even artefact distributions show a notably gentler slope with a higher radius value of maximum aggregation. Biased artefact distributions result in H-values which drop below zero at comparatively lower distances, indicating that separation into clusters starts earlier. The same occurs for evenly distributed artefacts at higher ϵ-values.

This underlines the potential of artefacts to both aid and hinder intra-cluster variability.

**Intra-cluster variability**. In simulations not influenced by artefacts, all agents within a cluster have approximately the same worldview. In simulations integrating artefacts, however, agents within a cluster may hold a spectrum of 𝐱-values bounded by artefacts.

We characterize this behaviour using the following relation R on {1,…,N}:


(4.4) $$iRj\;⟺\;j\in N_{i}^{\beta }(x^{t}).$$

Under the assumption of symmetry, this is reflexive but not transitive, and therefore not an equivalence relation. We introduce a closure with respect to transitivity and define the cluster relation by


(4.5) $$i∼j\;⟺\;\exists k_{n}\subset \{1,\ldots ,N\}\text{ such that }\;\;iRk_{1}\ldots Rk_{n}Rj.$$

**Definition 4.1**. *We define the cluster associated to agent i as*:


(4.6) $$c_{i}=\{j\in \{1,\ldots ,N\}|\;i∼j\}.$$

*If additionally*


(4.7) $$\begin{array}{ccc} & \exists k,l\in c_{i}:|\alpha_{k}-\alpha_{l}|>0 & \end{array}$$

*holds, we refer to cluster $c_{i}$ as an α-ladder cluster*.

The α-ladder cluster appears in a certain form.

**Proposition 4.1**. *On convergence of the model, when $A_{ag,ag}$ is symmetric, an α-ladder cluster is bounded by and spread between at least two artefacts*.

*Proof*. Let $\bar{x}=(\bar{\alpha },\bar{\beta })$ be an equilibrium of equation (3.6). Therefore for any k


(4.8) $$\begin{array}{ccc} & {\bar{\alpha }}_{k}=AVG\left(\{{\bar{\alpha }}_{j}^{t}|\;j\in N_{k}^{\alpha }(x^{t})\}\right) & \end{array}$$

holds. Let then c⊂{1,…,N} be a cluster according to equation (4.7). By definition, there exist indices i,j∈c such that


(4.9) $$\begin{array}{ccc} & i∼j\wedge |{\bar{\alpha }}_{i}-{\bar{\alpha }}_{j}|>0. & \end{array}$$

As i∼j, there must exist a sequence $k_{n}\subset \{1,\ldots ,N\}$ such that $iRk_{1}\ldots Rk_{n}Rj.$ However, as ${\bar{\alpha }}_{i}\neq {\bar{\alpha }}_{j}$ there must exist at least two indices $m,n\in \{i,k_{1},\ldots ,k_{n},j\}$ where


$$mRn\wedge {\bar{\alpha }}_{m}\neq {\bar{\alpha }}_{n}.$$

Without loss of generality let ${\bar{\alpha }}_{m}>{\bar{\alpha }}_{n}$. The α-update (3.5) assigns to ${\bar{\alpha }}_{m}$ the average α values of all agents in $N_{m}^{\alpha }(\bar{x})$. As the model converges to an equilibrium, ${\bar{\alpha }}_{m}$ must not be changed by the update. Given that ${\bar{\alpha }}_{n}$ is smaller than ${\bar{\alpha }}_{m}$, there must exist a $b\in N_{n}^{\alpha }(\bar{𝐱})$ where ${\bar{\alpha }}_{b}>{\bar{\alpha }}_{m}$.

If b is an artefact, the proof is complete. If b is an agent, then as $A_{ag,ag}$ is symmetric and


$$b\in N_{n}^{\alpha }(\bar{x})\;⟺\;n\in N_{b}^{\alpha }(\bar{x})$$

holds. The process can therefore be repeated for ${\bar{\alpha }}_{b}>{\bar{\alpha }}_{m}$. As the number of agents is finite, this must necessarily lead to an artefact bounding α, as it is not updated by averaging.∎

In this bounded confidence model, this proves that artefacts can lead to increased variability of opinions and values within a cluster. See the appendix (Discrepancy between values and opinion) for details on inter-agent variability between α and β.

## 5. Discussion

Our results underline the potential of artefacts to increase as well as to reduce diversity in worldviews.

Their impact largely depends on how they are distributed across a society as well as on the general inclination towards considering diverging opinions within a society. This openness to different views is codified by ϵ. Greater openness, indicated by a higher ϵ-value, comes with a greater potential for artefacts —or human actors—to enact influence. On the flip-side, low levels of openness deeply entrench the agent’s worldview, restricting their willingness to enter dialogue and debate with those differently minded.

In the structurational view, technological artefacts are a medium of human action, facilitating and constraining behaviour through interpretative schemes, facilities and norms. When large-scale applications for opinion formation are developed to represent a subset of the population in world-view, they have the potential to reinforce institutional properties (if inline with the views and wants of artefact developers) or of transforming them (if developers hold minority views). When the power to create and spread artefacts is held mainly by people of a particular viewpoint, they may be able to shift the systemic view in their favour. Institutional structures of domination generally grant power and resources to shape artefacts to those benefiting from the status quo. Such an imbalance may result in a feedback loop, fortifying the position of the powerful and shifting worldview distributions on a societal level, and leading to institutional consequences further entrenching existing structures of signification, domination and legitimation.

When artefacts unevenly represent society, the populated worldview space is reduced asymmetrically with increasing openness, leading to a loss of agents holding central or moderate views (columns biased and shifted). This results in polarization, forcing opinions further apart. The large majority of opinions is, however, biased towards the artefact viewpoints. This is evident as well when artefacts are evenly distributed, resulting in the symmetric reduction of agents holding extreme views (figure 2, column ‘even*’*).

Such an effect may be an unintended byproduct. Artefact designers may have the best intentions, holding themselves up to high ethical standards of transparency and oversight and attempting to minimize the influence of their own opinions. However, artefacts inherently represent the designers’ worldview. In the case of VAAs, for example, any attempt to reduce bias in recommendation still acts in the rigid value framework of social choice theory [18,19]. If bias is indeed intended, artefacts are potential enablers of exploitation. For example, social media was host to propaganda campaigns and targeted manipulation, which had been engineered to spread and to enact influence [29].

At the same time, artefacts may provide stable anchors, upholding the diversity of information, opinions and values that an agent is confronted with. This is evident when artefacts are few, located also at the extreme ends of the value space, and the loss of diversity is dispersed throughout the worldview space (column ‘few’).

In summary, structures of domination during the development of an artefact have the potential to affect views on a large scale during artefact use, and by extension the institutional structures guiding human action. If power asymmetries systematically benefit groups with greater resources upholding minority viewpoints, these structures may be fortified in subsequent iterations of technological innovation.

In particular, designers and developers tend to hold greater power over an artefact than its users and often represent different subgroups of a population, interacting with the artefact according to different goals, propagating influence to users further along the artefact lifecycle.

Negative externalities such as increased homogeneity of worldviews or polarization and the entrenchment of structures of domination call for the regulation and monitoring of artefact influence to ensure outcomes are societally beneficial.

These two stages in the artefact lifecycle, *artefact design* and *artefact use*, require different treatment for the regulation of minority influences. Interventions can be *substantive*, targeting the content or quality of an artefact, or they can be *procedural*, focusing the methods and processes. At the design stage, substantive interventions such as ethics guidelines may modify how the designers’ values are embodied in artefacts. Procedural interventions, such as introducing participatory processes and including stakeholders from diverse population groups, may target which values are embedded in artefacts. At the utilization stage, substantive interventions such as content moderation on social media may affect which values are propagated. Procedural interventions such as the increase of communication friction may mitigate to what extent values are spread.

The formal evaluation of non-epistemic requirements is a continuing challenge [1,2,6–8]. Social concepts such as fairness or trustworthiness may have diverging definitions in between different social groups or use cases, or no valid definition at all [2]. The substantive evaluation of artefacts requires a formalization of such concepts, and is itself influenced by institutional structures of domination. Procedural evaluation allows the intentional acknowledgement and mitigation of power imbalances.

There is also a danger to legislatory intervention. Pinch and Bijker [30] describe the life cycle of technology adopted in social context. In particular, the social groups concerned with an artefact define the core problem that an artefact is to— and may fail to—solve. In this manner, conflicting formal definitions can co-exist amongst different social contexts, with user choices implicitly prioritizing some definitions over others. In a pseudo-evolutionary process, this social group, through consumer choices, supports those artefacts embodying values close to theirs and so prioritizes the concepts of importance to their respective context.

An intervention by legislators or other powerful entities discontinues and overpowers the implicit selection and integration entailed in this process. This imposition may lead to developments that do not represent the values of a society. To avoid the disruption of value selection and development, any regulatory effort must consider the actors and their respective social contexts throughout the artefact design and life cycle.

We therefore stress the importance of procedural interventions, and in particular of participatory methods, for artefacts of public interest. The inclusion of diverse stakeholders situates an intervention socially, distributing power across a wider sample of the population and so mitigating the entrenchment of structures of domination. While the need for evaluation may be imposed legislatively, the definition and implementation should be left to those affected by an artefact.

**Limitations and outlook**. Within our model we make several simplifications and reductions of reality. While necessary to evaluate a question formally, this poses a limitation in terms of representatives. We use a static value of ϵ which is equal across agents and the agents’ neighbours. Similarly, we assume static social connections according to a predefined network. This precludes the possibility of agents getting to know new viewpoints on the fly. Additionally, we assume that a person’s opinions and values are consistent and do not change without cause. We also reduce one’s worldview into a position on a two-dimensional space. This representation may not do justice to their real complexity. Similarly, the interaction between values and opinion may not be one-dimensional or linear, and may not develop at the same rate. Finally there are theoretical counter-arguments to the concept of value-laden technology that our paper is built on [11,31,32].

Several important questions and goals for future research remain. First, while artefacts in our model are static, technology can be modified continuously by agents who will be influenced by the institutional properties present at the time. Our paper presents only a cross-section of this feedback loop between human agents, technology and institutional properties. Second, the reach of human agents and technological artefacts is not necessarily disconnected from structures of domination. Often, those individuals who are best connected also have more resources available to shape technological artefacts. The motivation to create artefacts may be affected by one’s social neighbours and their views. Investigating these interactions is integral to a greater understanding of longer-term dynamics.

## Data Availability

The code used to run the simulations is available on Github: https://github.com/ethz-coss/artefacts-and-worldview.

## Footnotes

^1^
We encourage the reader unfamiliar with the concept to explore and to critically examine the VAA for the 2024 EU elections (https://euandi.eu/) for the democratic values it champions.

## Appendix A

### Discrepancy between values and opinion

In the two-dimensional case, artefacts lead to an equilibrium where $\alpha_{i}\neq \beta_{i}$. This is a factor of the influence term λ, as evident when investigating under which circumstances $\alpha_{i}=\beta_{i}$:

$$\begin{array}{rl} \alpha_{i} & =AVG\;\left(\left\{\alpha_{j}|\;j\in N_{i}^{\alpha }(x)\right\}\right)\\ & =AVG\;\left(\left\{\alpha_{j}|\;j\in N_{i}^{\beta }(x)\right\}\right)+\left(AVG\;\left(\left\{\alpha_{j}|\;j\in N_{i}^{\alpha }(x)\right\}\right)-AVG\;\left(\left\{\alpha_{j}|\;j\in N_{i}^{\beta }(x)\right\}\right)\right),\\ \alpha_{i}=\beta_{i} & =AVG\;\left(\left\{\beta_{j}|\;j\in N_{i}^{\beta }(x)\right\}\right)+(1-\lambda )AVG\;\left(\left\{\alpha_{j}|\;j\in N_{i}^{\beta }(x)\right\}\right)\\ & =AVG\;\left(\left\{\alpha_{j}|\;j\in N_{i}^{\beta }(x)\right\}\right)+(1-\lambda )AVG\;\left(\left\{\alpha_{j}|\;j\in N_{i}^{\beta }(x)\right\}\right)\\ & =AVG\;\left(\left\{\alpha_{j}|\;j\in N_{i}^{\beta }(x)\right\}\right). \end{array}$$

Therefore, $AVG\left(\left\{\alpha_{j}|\;j\in N_{i}^{\alpha }(x)\right\}\right)=AVG\left(\left\{\alpha_{j}|\;j\in N_{i}^{\beta }(x)\right\}\right)$, meaning that the average alpha value for i’s neighbours is the same whether artefacts are included or not.

Consequently, if α=β, removing the artefacts leaves the equilibrium intact.
